# Antibiotic resistance pattern of multidrug-resistant Enterobacterales from a rectal surveillance study in northern Nigeria

**DOI:** 10.1093/inthealth/ihaf026

**Published:** 2025-03-25

**Authors:** Yahaya Yaqub, Joan Ejembi, Aliyu Aminu, Zainab Rabilu Daninna, Zainab Lamido Tanko, Nasiru Usman Ibrahim, Oduma Audu, Bawa Ega

**Affiliations:** Medical Microbiology Department, Ahmadu Bello University Zaria, Kaduna State, Nigeria; Medical Microbiology Department, Ahmadu Bello University Zaria, Kaduna State, Nigeria; Medical Microbiology Department, Bayero University Kano, Kano State, Nigeria; Medical Microbiology Department, Bayero University Kano, Kano State, Nigeria; Medical Microbiology Department, Kaduna State University, Kaduna State, Nigeria; Medical Microbiology Department, Aminu Kano Teaching Hospital, Kano State, Nigeria; Medical Microbiology Department, Ahmadu Bello University Teaching Hospital, Kaduna State, Zaria, Nigeria; Medical Microbiology Department, Ahmadu Bello University Teaching Hospital, Kaduna State, Zaria, Nigeria

**Keywords:** antimicrobial resistance, carbapenemase, ceftazidime-avibactam, ESBL, LMICs, surveillance study

## Abstract

**Background:**

Multidrug-resistant organisms (MDROs) are important in clinical practice worldwide. As whole genome sequencing (WGS) technologies are adopted, it is key to understand the nature of MDROs before the introduction of WGS in resource-poor settings.

**Methods:**

A hospital-based surveillance study was conducted in the largest referral health facility in northern Nigeria. A rectal swab sample was collected from each patient and samples were investigated for extended spectrum beta-lactamases and carbapenemase-resistant Enterobacterales (i.e. ESBL-PE and CRE, respectively). These MDROs were subjected to antimicrobial susceptibility testing and multiplex PCR. Statistical analyses were used to identify any associations between MDROs and selected antibiotics.

**Results:**

The prevalence of MDROs among participants (N=168) was 87.5% (n=147) for ESBL-PE and 4.2% (n=7) for CRE. All isolates were resistant to tetracycline and co-trimoxazole; however, most MDROs were susceptible to meropenem, ceftazidime-avibactam and fosfomycin (≥70%). bla_SHV_ (33.3%; n=49) was the predominant ESBL gene carried by the isolates, followed by combinations of bla_SHV_, bla_CTX_ and bla_TEM_. Although no carbapenemase genes were found, all CRE isolates had the bla_OXA-48_ gene, which may not be associated with phenotypic carbapenem resistance observed (χ^2^=0.056, p=0.81).

**Conclusions:**

Research utilising WGS and bioinformatics will elucidate more of the molecular landscape of MDROs in resource-poor settings.

## Introduction

Bacterial resistance to antibiotics surfaced almost immediately after the discovery of beta-lactam antibiotics in the 1940s. Thus began the race among humans trying to find new antibiotics on one hand and bacteria exhibiting a basic instinct of survival by demonstrating resistance to these antibiotics on the other.^1^ Bacterial resistance was first seen towards a single antibiotic agent, but this has since been replaced by the observation of many resistance patterns to different agents of different classes and modes of action in a simultaneous manner in these organisms.^2^ This characteristic of resistance to many antibiotic agents led to the concept of labelling organisms into various resistance categories by the Centre for Disease Control and Prevention (CDC) and European Centre for Disease Prevention and Control in 2012. Multidrug-resistance was assigned to organisms showing in vitro resistance to one antibiotic agent in ≥3 antibiotic classes, extensively drug resistance to those showing susceptibility to only one or two categories and pan drug resistance to those showing susceptibility to no agent in any category.^3^ Unfortunately this feature is not limited to a single bacterium or a group, but could be seen in virtually all known pathogenic bacteria.^4^

The Enterobacterales are one of the major causes of human diseases, causing both community- and hospital-acquired diseases. As Enterobacterales are commensals of the gut, this site provides them with a haven and thus a transit route through the hospital and the community.^5–7^ Production of enzymes that hydrolyse the agent is the most frequently utilised mechanism against the beta-lactam group of antibiotics by the Enterobacterales. These enzymes, collectively called the beta-lactamases, have evolved into several classes, of which the most problematic clinically are the extended spectrum beta lactamases (ESBL) and carbapenemases.^8^ The appearance of ESBL-producing Enterobacterales (ESBL-PE) in 1983 was a very serious setback to the antibiotic choices available to clinicians.^9^ This is due to the extent of resistance conferred by these enzymes that spares only the so called ‘antibiotics of last resort’ (i.e. carbapenems). Adding to this unfortunate situation is that carbapenems are quite expensive in Nigeria.^10^ The clinical report of carbapenemases-producing Enterobacterales (CPE) in 1993 further tilted the balance against humanity.^11^ These enzymes, ESBL and carbapenemases, have long been discovered to be mainly on plasmids and transposons, which makes them highly transferable among the *Enterobacterales.*^12^ ESBL-PE and CPE are invariably multidrug-resistant organisms (MDROs) as they usually harbour plasmids that additionally carry resistance genes against fluoroquinolones and aminoglycosides.^12,13^ It is important to note that CPE are a subset of a larger class of these organisms termed carbapenem-resistant Enterobacterales (CRE).^14^ Thus the usefulness of these frequently utilised alternatives is further limited.

Enterobacterales with MDRO characteristics were more often associated with hospitalised patients and the hospital environment, which was mainly due to the constant interaction between these organisms and the regular inevitable usage of antibiotics in the hospital.^1,6,9^ It is also an established fact that the epidemiological picture of resistant pathogens differs from country to country.^15^

Enterobacterales are responsible for a large number of hospital admissions worldwide, especially in the critically ill. The rise in the use of third generation cephalosporins has brought with it the problem of resistance to these antibiotics, especially among the Enterobacterales. The prevalence of these resistant organisms has been reported as between 10% and 70%, with ESBL-PE accounting for most of the burden. CPE were found to account for 2–7% of infection in the USA, Europe and Asia among ICU patients. In Greece, up to 25% of *Klebsiella* spp. isolates have been reported with carbapenemase resistance.^8^

Although the exact magnitude of the problem of resistant Enterobacterales may be difficult to establish in Nigeria, the finding of Okesola et al., regarding the prevalence of ESBL-PE isolates being 76.9% among clinical isolates in Oshogbo, is disturbing.^16^ There have also been reports of the recovery of CPE from Kano and Lagos.^17,18^

### Justification for the study

For the first time, in 2013, the CDC deemed it necessary to set threat levels to infectious agents as ‘urgent’, ‘serious’ and ‘concerning’ in decreasing order of importance, which is not in keeping with the recognised classification for infectious agents of bioterrorism. This was in response to the recognised increase in incidence of these MDR pathogens worldwide, and thus CPE and ESBL-PE were designated the threat levels ‘urgent’ and ‘serious’, respectively.^19^ The CDC thus recommended the regular surveillance of these MDROs with faecal colonisation as an important tool.^20^

Olusoga et al. found a faecal carriage rate of 15.5% (n=114) for ESBL-PE in southern Nigeria.^21^ Research on the faecal carriage of ESBL-PE in northern Nigeria and/or CPE carriage among humans in Nigeria, respectively, is scarce. However, there have been studies from other Third World countries such as Argentina,^22^ Pakistan,^23^ Ethiopia,^24^ Morocco,^5^ Madagascar^2^ and Cameroon.^25^

The objectives of this study were (i) to determine the prevalence of faecal colonisation by MDROs among inpatients at Ahmadu Bello University Teaching Hospital (ABUTH), Zaria; (ii) to identify bla_TEM_, bla_SHV_ and bla_CTX_ genes among these ESBL-PE isolates; (iii) to identify bla_KPC_, bla_NDM_, bla_VIM_, bla_IMP_ and bla_OXA-48_ genes among the CRE isolates; and (iv) to evaluate the in vitro susceptibility of these resistant organisms (i.e. ESBL-PE and CRE) to other antibiotic agents.

It has been well established that asymptomatic gastrointestinal colonisation by ESBL-PE and CPE is a prelude to infections associated with them.^26^ To the best of the authors’ knowledge, this is the first study looking at antibiotic resistance surveillance of Enterobacterales from rectal origin at the ABUTH, Zaria. These findings could be used as objective evidence to stimulate the management of the hospital on the need to strengthen infection, prevention and control measures. This study also provides objective information that will help in the rational choice of antimicrobial agents in the empirical therapy of serious infections due to ESBL-PE and CRE locally.

## Materials and methods

### Study area

ABUTH Shika-Zaria is a tertiary health facility in Kaduna state in the northwestern region of Nigeria. It has a 1000-bed capacity. It receives around 10 000 patients each year. As a referral centre, it also receives a large number of patients from all over the country.

The Medical Microbiology department of the hospital receives clinical samples for processing from the general outpatient and specialty clinics, all wards (medical, surgical, paediatrics, obstetrics/gynaecology and oncology), as well as ICU and Special Care Baby Unit (SCBU). It also receives samples from other healthcare facilities within Zaria.

### Study design

This was a cross-sectional study to describe the antibiotic resistance pattern of MDRO (i.e. ESBL-PE and CPE) isolated from rectal swabs of hospitalised patients at ABUTH, Zaria. It involved rectal swab sample collection from participants in the following wards: medical, surgical, paediatrics, gynaecology and oncology, ICU and SCBU. These samples were processed at the Medical Microbiology laboratory of ABUTH, Zaria, while molecular characterisation was performed at DNA laboratories in Kaduna.

### Sample size calculation

Sample size calculation was performed using the cross-sectional sample size calculator on Epi Info^TM^ 7 (USA). The power of 80%, two-sided 95% CI and 11% expected outcome in the unexposed^27^ were used. The total sample size was 168 for Kelsey's method.

### Inclusion criteria

Inclusion criteria consisted of patients admitted to wards (i.e. medical, surgical, paediatrics, gynaecology and oncology, ICU and SCBU).

### Exclusion criteria

Exclusion criteria consisted of patients who were in the above categories but either had a colostomy or were on dialysis.

### Sampling technique

The sample size of 168 was divided across the seven units equally, leading to recruitment of 24 patients in each unit. Patients in odd-numbered beds were recruited during a single visit. Subsequently, new patients were enrolled if they were in odd-numbered beds. This process continued until the sample size was reached. However, in the ICU and SCBU, all patients were recruited if they had not been earlier recruited into the study from the referring ward. This was due to the scarcity of patients. The recruitment of patients and laboratory processing of rectal samples took 10 mo from January to November 2017.

### Ethical considerations

Ethical approval was obtained from the Ethics Committee of ABUTH, Zaria, before commencement of the study. Also, written informed consent was secured from every patient who participated in this study.

### Sample collection and transport

A labelled rectal swab was collected, using a double-stick Amies transport swab, from all consenting participants. The rectal swab samples were collected by gently inserting the swab 2.5 cm deep into the anal orifice while rotating it clockwise. The swab was then removed and immediately placed into an accompanying Amies transport media then taken to the laboratory for processing. This transport media is reported as good for the transport of Enterobacterales.^28^

### Primary isolation and screening

One of the swab sticks was cut and placed into a tube containing 5 mL of tryptic soy broth (TSB) and meropenem (10 μg) disk for CPE screening of Enterobacterales.^29^ The other swab stick was streaked for discrete colonies on a half-plate of Brillance ESBL agar (Oxoid, UK). These were then incubated at 35–37°C for 18–24 h. The swab for carbapenemase screening (i.e. from the TSB and meropenem [10 μg] disk) was then inoculated on MacConkey agar and streaked for discrete colonies. The MacConkey agar was read after 24 h and any growth was considered carbapenem resistant and subjected to Gram staining. Likewise, growth seen on the Brillance ESBL agar (i.e. ESBL-positive isolates) was also subjected to Gram staining. Gram-negative bacilli isolates were subcultured onto nutrient agar, after which oxidase testing was performed on them. The oxidase test was performed by picking a colony of the isolate from the nutrient agar plate using a wooden stick and placing it on an oxidase strip (Oxoid, UK). An immediate appearance of purple discoloration was considered as positive and therefore not an *Enterobacterale*. Only oxidase-negative Gram-negative bacilli isolates (i.e. Enterobacterales) were subjected to further analysis for this study.

### Bacterial identification with microbact 24E system

Bacterial identification was performed using Microbact 24E system (Oxoid, UK) as per the manufacturer’s instructions. The Microbact computer-aided algorithm, which is based on an octal coding system, was used to arrive at the identity of the Enterobacterales.^30^

### Antimicrobial susceptibility testing

Antibiotic susceptibility testing was carried out of all the isolates to the following antibiotic agents in accordance with the clinical laboratory standard institute (CLSI M100-S26) document for disc diffusion: ceftazidime-avibactam (14 μg), piperacillin-tazobactam (100/10 μg), gentamicin (30 μg), amikacin (30 μg), ciprofloxacin (5 μg), trimethoprim-sulfamethoxazole (i.e. co-trimoxazole) (1.25/23.75 μg), tetracycline (30 μg), chloramphenicol (30 μg), meropenem (10 μg), fosfomycin (50 μg) and nitrofurantoin (300 μg).^31^

### Phenotypic confirmation of ESBL production

Modified double disk synergy was used. This was performed by flanking amoxicillin-clavulanic acid (20/10 μg) disk with disks of ceftazidime (30 μg), ceftriaxone (30 μg), cefepime (30 μg) and aztreonam (30 μg), each at 20 mm apart. This was in accordance with the CLSI M100-S26 document, to increase the chances of confirmation, because of the diversity of resistance enzymes belonging to ESBL. A distortion of zone between amoxicillin-clavulanic and any of the other four antibiotics confirmed ESBL production.^31^ All of the CRE isolates were subjected to ESBL confirmation as well and meropenem (10 μg).

### Phenotypic confirmation of carbapenemases production

Isolates that grew from the TSB were considered to be CRE and thus a modified Hodge test (MHT) was performed on them to confirm carbapenemase production, as enshrined in the CLSI M100-S26 document.^31^ Any isolate having a distorted zone (i.e. clover-leaf appearance) around the test organism compared with the positive control was considered to be MHT-positive.

### Quality control strains used for ESBL-PE and CPE screening and confirmation

The following reference strains were utilised for this research: *Escherichia coli* ATCC 25922, *Klebsiella pneumoniae* ATCC—BAA 1705 (positive MHT control) and *K. pneumoniae* ATCC—BAA 1706 (negative MHT control).

### Primers, amplification and gel electrophoresis

Appropriate primers were used to target TEM, SHV and CTX genes^32^ from the ESBL-PE and KPC, NDM, VIM, IMP and OXA-48 genes^33^ from the CRE. Table 1 and Table 2 show the primer mixes, which have been validated for multiplex runs, that were used in this study.^32,33^ A negative control was also used for control of the multiplex PCR reactions. A 100–1000 bp molecular ladder was used to read the amplicon sizes yielded.

**Table 1.** ESBL target enzyme, primers and amplicon size used in this study

Target	Primer sequence (5′-3′)	Expected amplicon size (bp)
TEM	GGTCCTCCGATCGTTGTCAG (F) GCACGAGTGGGTTACTCGA(R)	404
SHV	CGCCTGTGTATTATCTCCCT(F) CGAGTAGTCCACCAGATCCT (R)	294
CTX	CGCTGTTGTTAGGAAGTGTG (F) GGCTGGGTGAAGTAAGTGAC (R)	754

The reference is Roschanski et al.^32^

**Table 2.** Carbapenemases target enzyme, primers and amplicon size used in this study

Target	Primer sequence (5′-3′)	Expected amplicon size (bp)
KPC	ATGTCACTGTATCGCCGTCT (F) TTACTGCCCGTTGACGCCC (R)	785
NDM	ACTTGGCCTTGCTGTCCTT (F) CATTAGCCGCTGCATTGAT (R)	603
VIM	TGTCCGTGATGGTGATGAGT (F) ATTCAGCCAGATCGGCATC (R)	437
IMP	ACACGGCTTAGTAGTGCTTG (F) GGTTTAACAAAACAACCACC (R)	387
OXA-48	ATGCGTGTATTAGCCTTATCG (F) CATCCTTAACCACGCCCAAATC (R)	265

The reference is Bogaerts et al.^33^

A multiplex PCR amplification reaction for the ESBL genes was carried out for the ESBL-PE isolates, while both ESBL and carbapenemase genes were searched for among the CRE isolates. Each reaction mixture consisted of 1 µl of each primer mix and 1 µl of extracted DNA added to 13 µl of heavy water to make up to 15 µl, then centrifuged. The tubes were placed in a thermocycler for the PCR reaction. An initial denaturation at 95°C for 5 s was followed by a 40-cycle denaturation at 95°C for 30 s, 57°C for 30 s and 72°C for 30 s. This was followed by a final extension at 72°C for 2 min.

The amplified products were subjected to electrophoresis in 1.5% agarose gel at 120 V for 1 h 15 min. Ethidium bromide was used as the fluorescent dye and a molecular marker was used to assess amplicon band size. The bands were viewed under ultraviolet light using Gel Doc 2000 Biorad, which was also used to capture the image.

### Data analysis

Data from each participant were entered into and cleaned in an Excel spreadsheet, followed by statistical analyses in Stata 14 (StataCorp LP, College Station, TX, USA).

The proportions of ESBL and CRE carriage were calculated. The proportion of resistant enzyme among isolates was also calculated. The proportion of genes for ESBL and cabapenemase resistance carried by the isolates (i.e. individually and in combination) was calculated. The proportion of resistance to antibiotic among isolates was calculated. The significance of the observed difference between the proportions of resistance of these antibiotics compared with that of meropenem was calculated using the χ^2^ test.

All p values for this study were two-sided and p≤0.05 was considered significant. All CIs were 95% and were only considered good if they did not include the null figure (i.e. 0 for z and χ^2^ tests and 1 for OR) and not wide from the observed values.

## Results

The overall prevalence for rectal carriage of MDROs among participants (N=168) of this study was 87.5% (n=147) for ESBL-PE and 4.2% (n=7) for CRE. No isolate was a CPE phenotypically. Most of the isolates in the current study were *E. coli* (83%, n=139). The rest consisted of *K. pneumoniae* and *Enterobacter aerogenes*: 15% (n=25) and 2% (n=4), respectively. All CRE were *K. pneumoniae*, accounting for 28% (7/25) of the total *K. pneumoniae* found in this study.

### Antibiotics susceptibility pattern of the isolates

**Figure 1.**
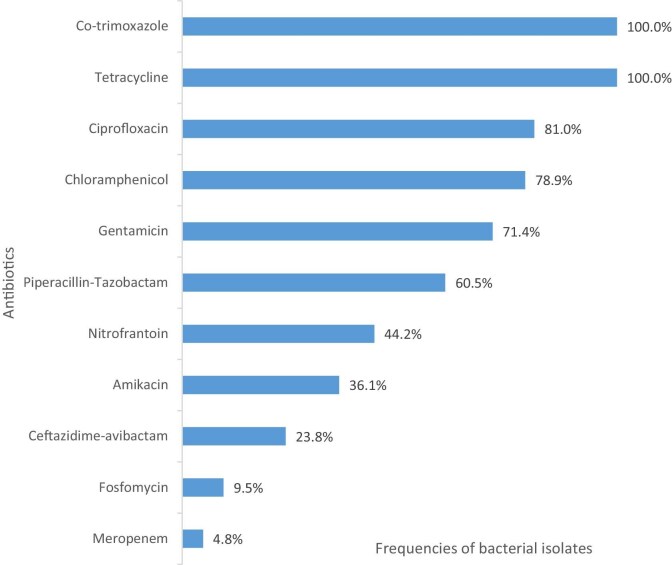
Antibiotic resistance pattern for all isolates (N=147) from this study.

In total, 74.1%, 81% and 78.9% of isolates were resistant to gentamicin, ciprofloxacin and chloramphenicol, respectively. Also, 9.5%, 4.8%, 23.8%, 36.1%, 60.5% and 44.2% of the isolates were susceptible to fosfomycin, meropenem, ceftazidime-avibactam, amikacin, piperacillin-tazobactam and nitrofurantoin, respectively. All of these isolates (i.e. 100%) were susceptible to tetracycline and co-trimoxazole (Figure 1).

### Comparing the significance of the difference in proportion of resistant isolates of other antibiotics with meropenem

The susceptibility of the isolates to the antibiotics used in this study was compared with the susceptibility of the same isolates to meropenem to demonstrate the reliability of other antibiotics as alternatives to meropenem (Table 3).

**Table 3.** Comparison of the frequencies of antibiotic resistance of the isolates to meropenem against other antibiotics used in this study

	Resistant isolates		95% CI		Comparison with meropenem resistance	
	No.	Freq. (%)	Lower	Upper	χ^2^ value	p
Meropenem	7	4.8%	1.9%	7.6%	Control	"—
Fosfomycin	14	9.5%	5.6%	13.5%	2.55	0.1100
Ceftazidime-avibactam	35	23.8%	18.0%	29.6%	21.9	<0.001
Amikacin	53	36.1%	29.6%	42.5%	44.5	<0.001
Nitrofrantoin	65	44.2%	37.5%	50.9%	61.8	<0.001
Piperacillin-Tazobactam	89	60.5%	53.9%	67.2%	103.8	<0.001
Gentamicin	105	71.4%	65.3%	77.5%	147.8	<0.001
Chloramphenicol	116	78.9%	73.4%	84.4%	165.8	<0.001
Ciprofloxacin	119	81.0%	75.6%	86.3%	174.1	<0.001
Tetracycline	147	100.0%	98.2%	100.0%	***	<0.001
Co-trimoxazole	147	100.0%	98.2%	100.0%	***	<0.001

***All resistant isolates (i.e. n=147) were resistant to this antibiotic, thus a comparison cannot be made.

### Pattern of resistance genes from isolates by PCR

**Figure 2.**
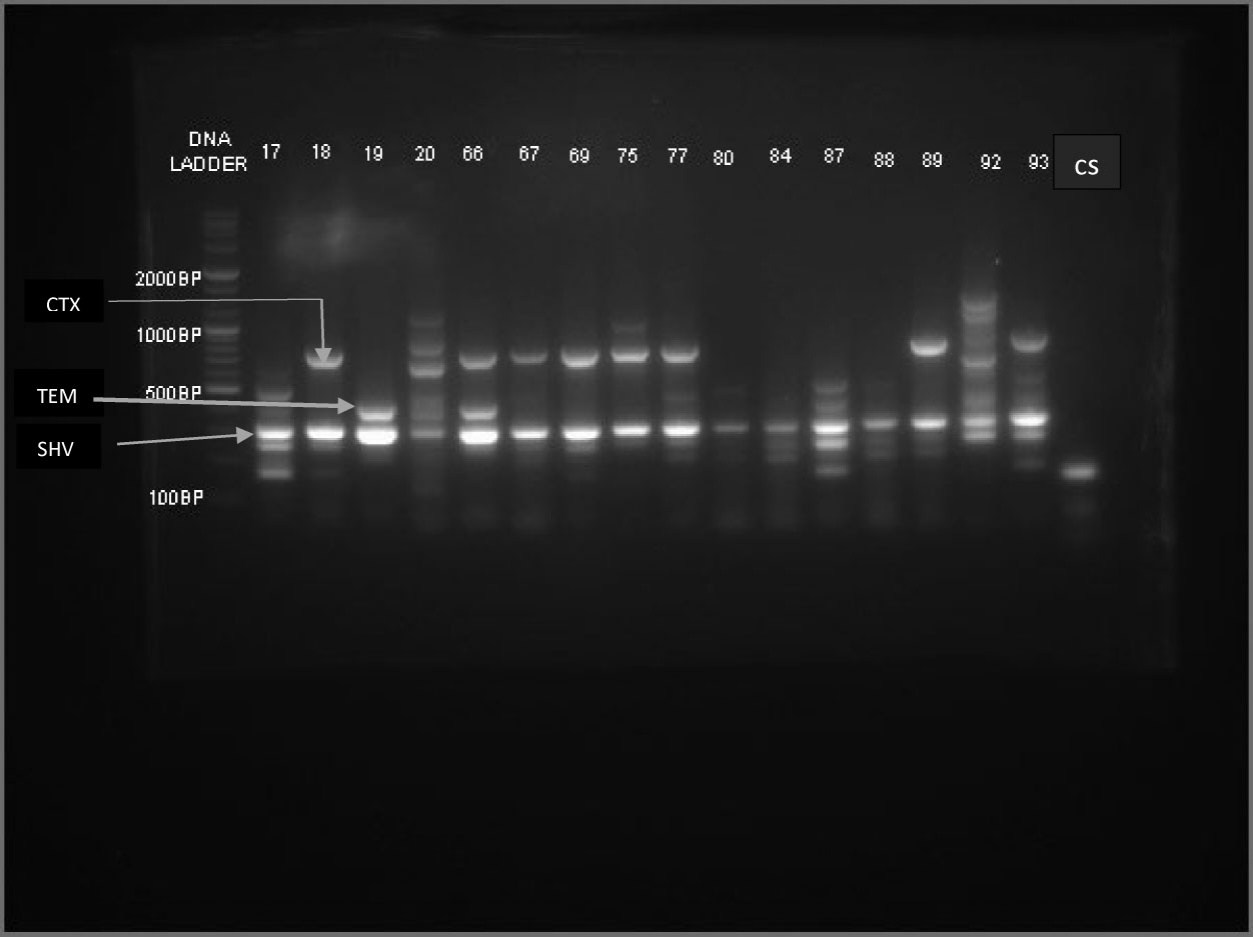
General snapshot showing the resistance enzyme pattern from ESBL-PE in this study. Note: 1. The numerous SHV bands. 2. cs=control strain (*E. coli* ATCC 25922). 3. SHV=294 bp, TEM=404 bp and OXA-48=265 bp.

**Figure 3.**
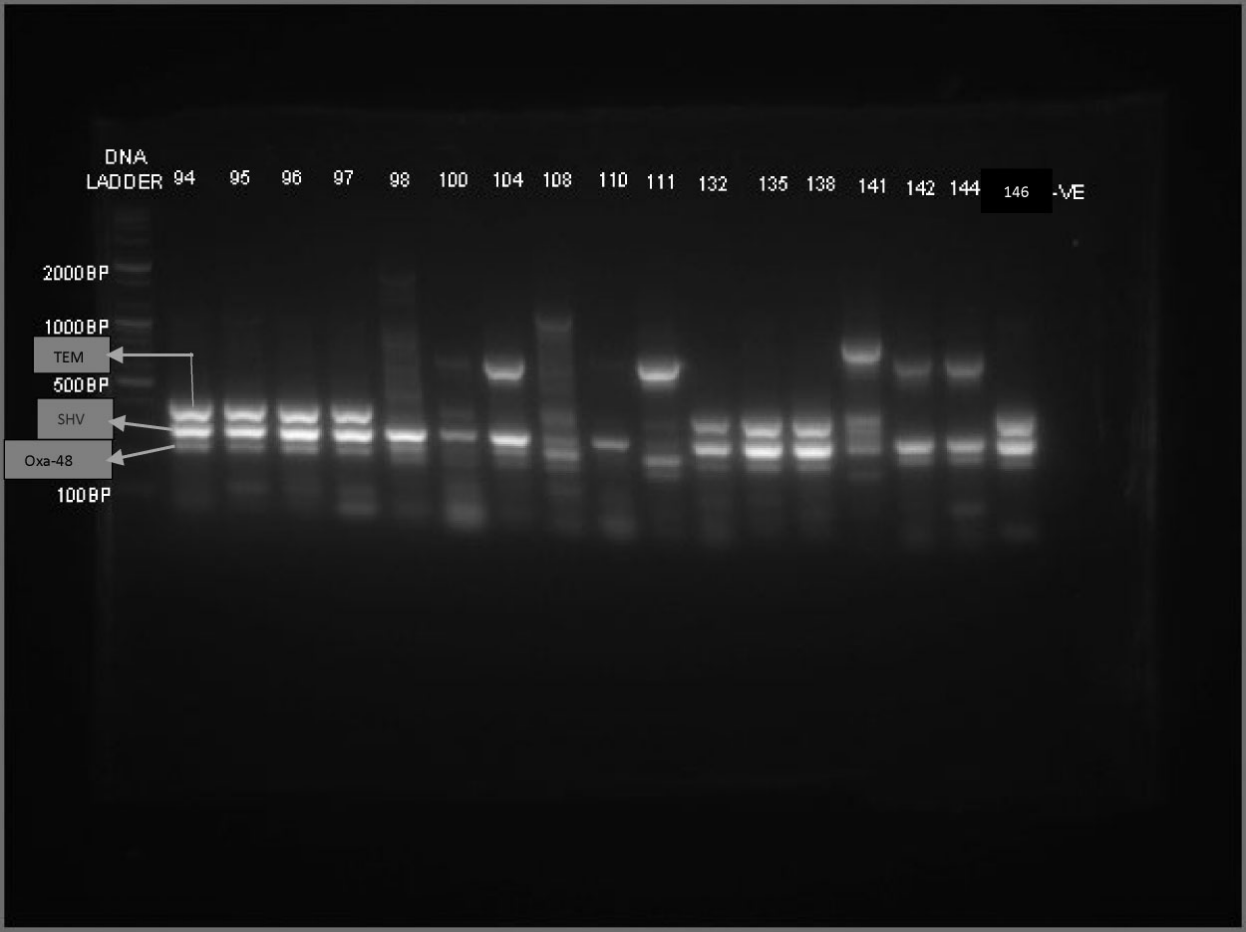
PCR findings of CRE isolates and other observations of note from this study. Note: 1. The similar enzyme pattern of 94, 95, 96, 97, 135 and 138 (they were all CRE and *K. pneumoniae*) had OXA-48, SHV and TEM bands; 132 and 146 had similar bands but were not CRE and were *K. pneumoniae* and *E. coli*, respectively. 2. -ve is negative control (i.e. blank). 3. SHV=294 bp, TEM=404 bp and OXA-48=265 bp.

bla_SHV_ gene was found alone in 49 (33.3%) isolates. There were, however, varying degrees of combinations between these genes (Table 4 and Figure 2). All CRE isolates (n=7) were *K. pneumoniae.* Surprisingly, none of these isolates were positive for the phenotypic MHT. This was mirrored by the PCR studies, as no bla_KPC_, bla_NDM_, bla_VIM_ or bla_IMP_ genes were found, but all exhibited bla_OXA-48_ (Figures 2 and 3).

**Table 4.** Proportion of resistance enzyme genes among ESBL isolates by PCR found in this study

Amplified genes of resistance enzyme	Number of isolates	Proportion (%)
bla_SHV_ only	49	33.3
bla_CTX_ only	1	0.7
bla_TEM_ only	0	0
bla_SHV_ and bla_CTX_ only	37	25.2
bla_SHV_ and bla_TEM_ only	23	15.6
bla_CTX_ and bla_TEM_ only	0	0
bla_SHV_, bla_CTX_ and bla_TEM_ only	37	25.2
Total	147	100

Note: only bla_OXA-48_ carbapenemase (4.2%, n=7) was found in this study.

## Discussion

Although very different, the faecal colonisation rates for ESBL-PE and CRE of 87.5% (p<0.001, 95% CI 81.53 to 92.1%) and 4.8% (p=0.022, 95% CI 2.0 to 9.1%) (N=168), respectively, were surprising. An earlier study with a similar design but a smaller sample size (n=77) in Morocco found lower ESBL-PE (43%) but higher CRE (10%) rectal carriages.^6^ Thus, despite the smaller sample size in the Morocco study, the ESBL and CRE findings were similar. Wang et al. reported a prevalence of 5% for CRE (n=1135) that were not CPE in 2015 from Taiwan, which was similar to the findings of the current study.^34^ The Taiwan study also revealed findings of CRE that fall within the 95% CI (i.e. 2% to 9%) from the current study. The finding that a large majority of the isolates were *E. coli* (83%) is similar to what Villar et al. found in an earlier study.^22^ However, this should be considered with caution as this may simply be a reflection of the normal composition of the enteric bacteria.

A striking find, however, was that all CRE were *K. pneumoniae*, and all were confirmed as ESBL (i.e. also grew on Brillance chrome agar) as well. All of these CRE isolates were also resistant to quinolone, aminoglycosides, ciprofloxacin, tetracycline and trimethoprim-sulfamethoxazole. It has been previously reported that concurrent resistance to carbapenem, quinolone and aminoglycosides was strongly associated with overexpression of efflux pump.^35^ Thus it could be that another mechanism of resistance (such as efflux pump) might have been employed by these isolates other than the production of hydrolysing enzymes. A study in Korea found a similar characteristic of CRE isolates from the ICU.^36^

Only fosfomycin can be inferred to be a reliable alternative to meropenem as it is the only antibiotic found not to have any significant difference in antibiotic susceptibility among the isolates tested in this study (Table 3). The pattern of resistance towards a novel antibiotic such as ceftazidime-avibactam, which is anecdotally yet to be available for clinical usage in northern Nigeria, is particularly disturbing. A recent study reported that porin mutation and overexpression of ESBL were associated with observed resistance where drug exposure is yet to occur, which may explain the observations in the current study.^37^ This may be an important surveillance focus when eventually it begins to be used in our context as studies have demonstrated the utility of ceftazidime-avibactam for the treatment of complicated intra-abdominal infections and urinary tract infections (UTIs) associated with Enterobactorales.^37,38^ The role of fosfomycin in the treatment of UTIs has been gaining interest recently.^39^ Nitrofurantoin was reported to be at least equal to other alternatives in treating UTIs by a recent systematic review.^40^

bla_SHV_ was the predominant ESBL gene carried by the isolates, followed by bla_CTX_ and bla_TEM_, respectively, in this study (Table 4). This was not different from the findings in Uganda where bla_SHV_ was also the most prevalent.^41^ Also in this study none of the CRE exhibited any bla_KPC_, bla_NDM_, bla_VIM_ or bla_IMP_ genes, which may not have been unrelated to the findings of two independent studies from Uganda, where bla_OXA-48_ and bla_VIM_ seem to be the predominant genes expressed.^42,43^ Although bla_OXA-48_ was expressed by these CRE isolates, this finding should be viewed with caution as this may just have been a chance occurrence (χ^2^=0.056, p=0.81).

Perhaps more information may have been yielded if other molecular characteristics of these isolates, such as strain type or genome profile, had been investigated. It was also important that no other mechanisms of resistance were investigated for beyond the production of the hydrolysing enzymes. Perhaps this would have given more resolution to the molecular rapport of the MDROs found in this study. It is important to appreciate how the molecular landscape of these MDROs in our environment (and other contexts) has changed since the COVID-19 pandemic. As diagnostic microbiology rapidly evolves to incorporate newer technologies such as whole genome sequencing and bioinformatics into routine use in sub-Saharan Africa, it is important to conduct contemporary research to gain a better understanding of the antibiotic resistance problem in sub-Saharan Africa.^44^ This would surely aid in developing context-related policies that will yield efficient and effective solutions to the problem of antimicrobial resistance.

## Data Availability

The data are provided in the article.
